# A Sentence Repetition Task for Catalan-Speaking Typically-Developing Children and Children with Specific Language Impairment

**DOI:** 10.3389/fpsyg.2017.01865

**Published:** 2017-10-30

**Authors:** Anna Gavarró

**Affiliations:** Departament de Filologia Catalana, Universitat Autònoma de Barcelona, Barcelona, Spain

**Keywords:** specific language impairment, sentence repetition task, Catalan

## Abstract

It is common to find that so-called minority languages enjoy fewer (if any) diagnostic tools than the so-called majority languages. This has repercussions for the detection and proper assessment of children with Specific Language Impairment (SLI) brought up in these languages. With a view to remedy this situation for Catalan, I developed a sentence repetition task to assess grammatical maturity in school-age children; in current practice, Catalan-speaking children are assessed with tests translated from Spanish, with disregard of the fact that the markers of SLI may differ substantially from one language to another, even between closely related languages. The test proposed here is inspired by SASIT [School-Age Sentence Imitation Test – English], designed for English by Marinis et al. (2011); some of the constructions targeted are challenging in a subset of languages, but not others, and are included because they are indeed affected in Catalan SLI; other constructions appear to be disrupted universally. The test involves canonical SVO sentences, sentences with third person accusative clitics (known to be problematic in Catalan SLI, but not in Spanish), passives, wh- interrogatives, subordinate clauses, subject and object relatives and conditionals. The test was administered to thirty typically developing 6- and 7-year-olds (as reported in Gavarró et al., 2012b), and five children diagnosed with SLI (mean age 10;7). The results of the task were scored under two systems: (i) identical vs. non-identical repetition and (ii) identical, grammatical and ungrammatical repetition, with detail regarding the error type. The results for typically developing and SLI children showed differences between the groups: identical repetition was found in 88.9% of cases for typically developing children but only 48% for SLI children. Ungrammatical productions were higher for the SLI group, and so were grammatical but different repetitions, a trend which was found in every child individually. The results are compared to those available in the literature for similar languages and I discuss the impact of grammatical variation in language performance, in both typical and impaired development.

## A sentence repetition task for catalan: motivation and goals

Specific Language Impairment (SLI) is a developmental deficit affecting spoken language in the absence of hearing impairment, neurological damage or intellectual disability (Leonard, 2003). It is well known that, in spite of stemming from a genetic condition (Stromswold, 2001; Bishop, 2002; Bishop et al., 2006), SLI manifests itself differently in different grammars. One classical example of such variation is the production of optional infinitives beyond the age at which these constructions disappear in typically-developing (TD) children, between ages 3 and 4. Optional infinitives are thus a reliable marker of SLI in English (Rice et al., 1995; Rice and Wexler, 1996). It is also known that optional infinitives are confined to non-null subject languages, both in TD and SLI children; in languages with null subjects, such as Greek, Italian and Catalan, optional infinitives are not generally found in child production (see Guasti, 2017 for a review of TD development). As a consequence, lack of finite inflection is not a universal marker; this has been widely shown, for example, in Italian (Leonard et al., 1992; Bottari et al., 1996 and others). On the other hand, in the null subject languages, omission of clitics and determiners have been reported to be reliable markers of SLI (see Bottari et al., 1998; Jakubowicz et al., 1998; Arosio et al., 2014). In general terms, markers of SLI are language-specific and therefore do not translate.

While tools to identify SLI have been developed for many languages, for other languages such tools are not available, and in some cases mere translations of tests designed for other languages are used, with detrimental consequences for diagnosis. To pursue the case of optional infinitives, if tense marking were to be used in a language like Italian, the vast majority of children with SLI would go undetected, judging by the results by Bottari et al. (1996). In addition, the lack of diagnostic tools is aggravated in the so-called minority languages and the problem goes well beyond SLI, as it affects other linguistic pathologies such as aphasia (for a recent review, see Fyndanis et al., 2017).

Together with the variable manifestations of SLI cross-linguistically, in an increasingly multilingual Europe there is a large population of early L2 learners whose linguistic level of attainment varies as a function of several complex factors. These factors comprise age of onset of acquisition (AoO), length of exposure (LoE), and socio-economic status (SES) (see Chiat et al., 2013 and references therein). For these L2 learners, language impairment is difficult to diagnose and may be misread as an effect of L2 acquisition. Likewise, L2 acquisition may be mistaken for language impairment. Both under-diagnosis and over-diagnosis are a source of concern for educators, clinicians and families^1^.

Tests to evaluate the linguistic competence of monolingual and bilingual children are scarce for many languages of the world, Catalan included (see Thordardottir, 2015 for a survey). In a large collaborative effort involving 30 countries, COST Action IS0804 “Language Impairment in a Multilingual Society: Linguistic Patterns and the Road to Assessment” set out to develop tools for the linguistic assessment of multilingual children and thus remedy the situation of multilingual children with SLI, an understudied and vulnerable group. The LITMUS [Language Impairment Testing in Multilingual Settings] battery of tests, still partly underway, is the outcome of this effort (see for further details, Armon-Lotem et al., 2015). Amongst the tools developed there were several sentence repetition tasks (SRT); this type of task has proven very effective in identifying children with SLI (Conti-Ramsden et al., 2001; Stokes et al., 2006; Bishop et al., 2009). SRTs vary in the way they are constructed and may as a consequence differ in the linguistic and cognitive abilities they measure (Crosnier, 2013), but researchers agree that grammatical reconstruction is necessary for sentence repetition to take place (Lust et al., 1996; Marinis and Armon-Lotem, 2015; Polišenská et al., 2015; see also Klem et al., 2015 for an overview of different views on exactly what SRTs measure). Even if e.g. short-term memory is recruited in sentence repetition, the task also reflects grammatical abilities.

The goal of this study is to present a sentence repetition task designed for Catalan, and to provide arguments for the inclusion of certain structures in the task and not others. The task was inspired by a similar one designed for English by Marinis et al. (2011), SASIT [School-age sentence imitation test – English], and developed as part of IS0804. However, the strength of the task presented here is that it is not a mere translation, but rather an adaptation grounded in the grammatical properties of Catalan and what is known about the manifestations of SLI in Catalan, which differ in many respects from English SLI. As illustrated below, even two closely related languages like Catalan and Spanish display different features in SLI, and thus the need for language-specific tools should not be underestimated (see Oetting et al., 2016 and references therein about the need for specific SRT for SLI in nonmainstream varieties of English).

What is the rationale behind the SRT proposed? Given that Catalan is a null subject language, one would expect an absence of optional infinitives and no general delay with finiteness. This is indeed what was found in a study by Gavarró (2012), where the spontaneous productions of two children with SLI at two stages in development were examined (data source: CHILDES). In the verbal production of these two children, aged 43 and 45 months in the first transcript, and 57 and 58 in the second, respectively, only 1.25% of optional infinitives were attested (computed over 556 verbal productions). On the other hand, in two studies of Italian-speaking children with SLI, even though optional infinitives were absent from the child productions, some problems with the production of third person plural verb morphology were encountered (Bortolini et al., 2002, 2006). The reason for this delay, which was specific to plural inflection, is not clear, though it is unlikely to have stemmed from the same source as optional infinitives, since only plurals were affected. Nonetheless, in light of these findings of Italian, canonical SVO sentences with compound or simple verb forms were kept in the SRT for Catalan as control items.

Much more characteristic of Catalan SLI is the omission of third person object clitics, exemplified in (1). This phenomenon was first identified for French SLI by Jakubowicz et al. (1998). Object clitic omission had been previously attested in TD children up to the age of 3–4 in French (Jakubowicz et al., 1996) and Italian (Schaeffer, 2000).

(1) Fico [e] aquí dins. (TD, Pep, 2;03,10)
  put-1s here inside
  “I put (it) here.”

Gavarró et al. (2010) argued that third person object clitic omission of the kind found in French and Italian was not universal, but rather due to language-specific checking operations—the very same checking operations that had been invoked to account for optional infinitives (Wexler, 1998). The prediction then was that if a language did not require such checking operations^2^ to take place in the derivation of object clitic constructions, clitics would not be omitted, but produced in an adult-like manner. This prediction was fulfilled, as Spanish-speaking children at age 2 did not omit clitics while Catalan-speaking children did so until age 3;6 (Gavarró et al., 2010). The prediction was further substantiated with results from Greek (Tsakali and Wexler, 2004), Romanian (Babyonyshev and Marin, 2006) and Spanish again (Elliot and Pirvulescu, 2016). Wexler (1998, 2014) argued that the checking mechanisms underlying clitic omission/optional infinitives were subject to maturation and that the optional stage finished during the third year of life, but persisted in children with SLI (see also Rice et al., 1995; Rice and Wexler, 1996).

Optional omission of third person object clitics is attested in SLI for French, Italian (Bortolini et al., 2002, 2006; Arosio et al., 2014) and Catalan (Gavarró, 2012). There is no evidence of clitic omission in Greek SLI in the studies by Terzi (2007) and Manika et al. (2011)—but see references therein reporting contradictory results. For Spanish, while the literature refers to deficits in clitic production (Bedore and Leonard, 2001), under closer scrutiny errors affect morphological markers (gender, number) rather than the production of the clitic itself, and the deficit is quantitatively minor compared to that seen in Catalan, French and Italian (see for recent results leading to the same conclusion, Martínez-Nieto and Restrepo, 2017). I conclude that clitic omission is not a marker of SLI in Spanish—or Greek. Uncontroversially, for Catalan it is a good candidate to serve as a clinical marker of SLI, as it has been shown to be in Italian (Arosio et al., 2014).

Though SLI child speakers of all three languages—French, Italian, and Catalan—show deficits in clitic production, the patterns of omission and replacement differ somewhat. The earliest results for French by Jakubowicz and colleagues showed that, when the target third person object clitic was not elicited, children with SLI produced alternatively a full DP (between 8 and 37.6%) or reflexive clitic (between 0.8 and 21.7%), or omitted the clitic altogether (between 4.7 and 66.4%). Later on Gavarró (2012) showed on the basis of experimental data from five Catalan-speaking children with SLI that these children failed to produce the target third person object clitic until age 5, at which point they produced a dative clitic instead, as illustrated in (2). By hypothesis, this dative clitic did not require the feature elimination that a third person object clitic required.

(2) … perquè la mare *li* pentina.
  because the mother clDAT combs
  “… because the mother combs his/her hair.”

More recently, Arosio et al. (2014) ran an object clitic elicitation task with 16 Italian-speaking children with SLI and found that they produced fewer target clitics than their age- and language-matched controls; instead, they produced more full DPs (35.94%), as well as some dative clitics (1.56%) or omitted object clitics altogether (8.98%). This varying performance that occurs when a third person object clitic is expected unravels which constructions are less problematic in each language. Arosio et al. (2014) found no difference as a function of age in their SLI group (aged 6;0 to 9;11). They pointed out, however, that a study with younger Italian children with SLI, that of Bortolini et al. (2006), showed higher rates of third person object clitic omission instead of full DP production. Therefore, the Catalan- and Italian-speaking children with SLI changed in performance over time (omission occurring first, replacement with a dative clitic or a full DP occurring with older children). For the purposes of the SRT it is clear that object clitics should be included, as they give rise to persistent problems in SLI.

For the same reasons that underlie third person object clitic omission, partitive clitics are omitted in early Catalan in TD children, but not beyond the age of 4 (Gavarró et al., 2006, 2011). By hypothesis, partitive clitic omission derives from the same underlying mechanisms as third person object clitic omission, and so under the same assumptions omission would be predicted for an extended period in SLI. Partitive clitics were therefore included in the task.

The SRT also included passive sentences. The comprehension of passive sentences is delayed in TD children, as studies in many languages over the years have attested (Maratsos et al., 1985 for English, Pierce, 1992 for Spanish, amongst others, see for a summary, Deen, 2011). Several hypotheses have been put forward to account for the delay (Wexler, 2004; Hyams and Snyder, 2005; Orfitelli, 2012, to cite only some of the recent ones), and most of them attribute the delay to some principle being subject to maturation; the maturational character of the emergence of passives is corroborated by the heritability effects discovered in twin studies (Ganger et al., 2005). Unlike third person object clitic production, however, passives are under-investigated in SLI, with only a few studies in English (van der Lely, 1996; Leonard et al., 2006; Marinis and Saddy, 2013). These studies confirm that passives are miscomprehended for an extended period in children with SLI. Given that Catalan shares the underlying syntax of passives with English and several other languages (raising of the object to the subject position), and that Catalan passives are misunderstood until age 6;6 by TD children (Parramon, 2016; Gavarró and Parramon, 2017), one would expect passives to be further delayed in Catalan SLI too. For this reason—despite their relative infrequency in the typical linguistic input received by small children—passives were included in the SRT.

Another structure that was included in the SRT is biclausal sentences. Biclausal sentences are part of the English SASIT (Marinis et al., 2011) and of the French LITMUS SR (Fleckstein et al., 2016). The rationale for including them is that complement clauses have been reported to be problematic for children with SLI in a number of studies, especially when the embedded clauses are finite, and that children with SLI produce fewer embeddings in their spontaneous productions than TD children (Scheidnes and Tuller, 2014). One particular case that has received much attention in the literature is that of relative clauses. There is an asymmetry in the comprehension of subject and object relative clauses, with subject relatives being better understood and produced than object relatives (at least in the head initial languages), as has been shown for many languages over the years (see Brown, 1971 for English, Labelle, 1990 for French, Arnon, 2005 for Hebrew, Arosio et al., 2009 for Italian, Gavarró et al., 2012a for Catalan, Girbau and Schwarz, 2007; Torrens, 2017 for Spanish). An analogous asymmetry is found in production. In a seminal paper, Friedmann et al. (2009) argued that the asymmetry could be accounted for in terms of intervention effects and Relativized Minimality, which would be stricter in childhood (until age 6 at least). Alternatively, processing analyses have been put forward; see for example Omaki and Lidz (2015) and Choe and Deen (2016). Without entering the discussion as to the nature of the asymmetry, Novogrodsky and Friedmann (2006) also show that object relatives in Hebrew SLI are more problematic than subject relatives and to a larger extent than in TD children, a finding that has been replicated for a number of languages (Delage et al., 2008 for French, Jensen de López et al., 2014 for Danish, Stavrakaki et al., 2015 for Greek, to mention a few). For this reason, repetition of subject and object relatives is included in the Catalan SRT, the expectation being that subject relatives will be less problematic than object relatives.

In a similar fashion, under the assumptions of Friedmann et al. (2009), wh- questions would be subject to stricter intervention effects in child grammar and the prediction therefore would be that, in a language with overt wh- movement such as Catalan, object wh- questions would be more taxing for children than subject wh- questions, an effect that would be prolonged in children with SLI. Amongst object wh- questions, in an experiment on Hebrew SLI by Friedmann and Novogrodsky (2011), *which* questions were more compromised than *who* object questions; this was captured by arguing that intervention effects hold only when the moved and the intervening element share the same features: in *which* questions, an NP specification, *which NP*, as opposed to *who*, as illustrated in (3).

(3) a. Et mi ha-xatul noshex?
  ACC who the-cat bites “Who is the cat biting?”
  b. Et eize kelev ha-xatul noshex?
  ACC which dog the-cat bites “Which dog is the cat biting?”

Again, these findings have been replicated in other experiments with children with SLI (Fleckstein et al., 2016). Note that the claim by Friedmann and Novogrodsky (2011) and Friedmann et al. (2009) is that the deficit in comprehension and production in SLI has to do with wh- movement and is structural, not derived from the presence of an embedded CP, contrary to the claims of Scheidnes and Tuller (2014).

On the basis of this background, the goals of the present paper are:

- To detail an SRT for Catalan with a strong motivation in our current knowledge of the grammatical characteristics of SLI in Catalan, and
- To provide results for TD children and children with SLI indicating that the SRT is sufficiently robust to meet the standard requirements of sensitivity and specificity (namely, the ability to reliably identify children with SLI and exclude children without it).

Before proceeding to a full description of the SRT task, let me mention that non-word repetition is a well-known task that discriminates between language-impaired and TD children (Bishop et al., 1996; Newbury et al., 2005). Still, the literature shows that the ability to repeat non-words may be impaired in only a subset of children with SLI (Bishop et al., 2006; Friedmann and Novogrodsky, 2008) and therefore cannot be the sole tool to identify children with SLI. Importantly, non-word repetition is spared in L2 although it is affected by language-specific differences between L1 and L2 (Polišenská, 2011), as the phonological words of the L2 may differ substantially from those of the L1 in phonological feature specifications, syllable structure and so on^3^.

## Method

Modeled on the similar instrument developed for English by Marinis et al. (2011), the proposed SRT for Catalan initially involved a total of 60 sentences classified into three levels of linguistic complexity, each level comprising 20 sentences. What is meant in Marinis et al. by complexity is not made explicit, and by the discussion in section 1 it is clear that some sentence types in level 1 may actually be very taxing in SLI in some languages.

In the current SRT each sentence ranges from six to eleven words and from seven to fifteen syllables (in the English SRT of (Marinis et al., 2011) the length of the items goes from seven to eleven words, and from eight to thirteen syllables)^4^. The proportion of different word lengths in the SRT was calculated: 0.23 of content words are monosyllabic, 0.45 are disyllabic and 0.31 are multisyllabic across the whole test; this matches quite closely the proportion in which these different word lengths are found in child speech (0.27 monosyllabic words, 0.53 disyllabic words, 0.19 multisyllabic words, see Guasti and Gavarró, 2003). Still, matching of experimental items was based on number of syllables per item, not on the length of content words, as in Marinis et al. (2011).

Frequency of the content words in the experimental items was taken into account, and the sentences include high frequency content words, based on the *Diccionari de freqüències* of Rafel i Fontanals (2006). Of the 118 content words in the SRT, 110 had a relative frequency between 1.985015 and 0.000511% in a corpus of 107,897 words found in the spoken language; this placed these 110 words in the task amongst the 8.8% most frequent words in the corpus. The remaining 8 words (including *mico* “monkey,” *cocodril* “crocodile,” *zoo* “zoo” and *pentinar* “comb”) were less frequent, but this result may stem from the fact that the corpus is not based on child and child-directed speech. The corpus of the vocabulary of 10 children in the CHILDES database by Serra et al. (2000) attested to the presence of most of them in child production in the period of 12–23 months. The frequency of words was matched across the three levels of the task.

Level 1 targets the following sentence types (in parentheses, the number of items for each sentence type):

- Canonical SVO sentences with an overt or a null subject, and a finite verb or a verb preceded by an inflected tense marker/verbal periphrasis (i.e., with additional functional vocabulary) (#8)
- Sentences with a third person object (accusative) clitic (#8)
- Sentences with a partitive clitic (#4)

Level 2 targets the following sentence types:

- Long passive sentences (#8)
- Wh- questions headed by *què* “what” or *quin* “which” (#8)
- Sentences with finite and non-finite complement clauses (#4)

Level 3 targets two sentence types, relative clauses and biclausal sentences with temporal dependencies between them:

- Subject relative clauses (#6)
- Object relative clauses (#10)^5^
- Sentences with a conditional clause (#4)

The sentences appear in the SRT in pseudorandom order, with sentences from levels 1, 2, and 3 intermingled, so that tiredness cannot especially affect sentences of level 3. Examples of each sentence type appear in Table 1.

**Table 1:** Contents of the STR proposed for Catalan.

**Structure type**	**Subtype**	**Example**	**Length in words and syllables**
Canonical SVO	Finite verb	*El gat perseguia la rata amunt i avall*. “The cat chased the rat up and down.”	6–8 words 11–13 syllables
Canonical SVO	Verbal periphrasis	*Ja pots portar els plats a taula*. “Now you can take the plates to the table.”	6–10 words 8–14 syllables
Accusative clitic		*La mare crida el nen i el banya*. “The mother calls the child and bathes him.”	5–11 words 9–13 syllables
Partitive clitic		*De pomes, n'he menjat tres*. “Apples, I have eaten three.”	6–8 words 7–11 syllables
Long passive sentences		*L'ós va ser caçat pel rei*. “The bear was hunted by the king.”	7–10 words 7–13 syllables
Wh- interrogatives	*qui/què* interrogatives	*Què van trobar ahir sota la neu?* “What did they find yesterday under the snow?”	6–8 words 8–11 syllables
Wh- interrogatives	*quin* “which” interrogatives	*Quina fotografia vas fer al parc?* “Which picture did you take in the park?”	6–8 words 9–11 syllables
Complement clause	Finite	*La mestra va decidir que aniríem al museu*. “The teacher decided that we would go to the museum.”	6–9 words 13–14 syllables
Complement clause	Non-finite	*Vam oblidar-nos de preparar l'esmorzar*. “We forgot to prepare breakfast.”	6–9 words 11–12 syllables
Relative clause	Subject	*El tren que ha sortit va a París*. “The train that left goes to Paris.”	8–10 words 8–15 syllables
Relative clause	Object	*L'ànec que el gat empaita no pot volar*. “The duck that the cat is chasing cannot fly.”	6–10 words 9–14 syllables
Conditional clause		*Els nens tindran un premi si netegen la classe*. “The children will get a prize if they clean the classroom.”	6–9 words 12–15 syllables

### Procedure

The procedure in the administration of this task is the same as that described in Marinis et al. (2011) and in all the LITMUS-SRTs, the only difference being that items were not recorded but read out by the experimenter, at a normal utterance pace and clearly articulated. The advantage of recording the items is arguable, as it disrupts communication between the child and the person carrying out the testing, while a live voice helps engage children in the task (Frizelle et al., 2017); for this reason recording of the sentences was avoided, even though this has the disadvantage of providing less homogeneous input to the children. The procedure is detailed in (4).

(4) – *Sentiràs unes frases i m'agradaria que repetissis exactament el que sents. No pateixis si no ho recordes tot, però mira de dir tot el que recordis, i de dir-ho clar*. [You will hear some sentences and I would like you to repeat them exactly as you hear them. Do not worry if you do not remember everything, but repeat everything you remember and do so as clearly as possible.]
  *Primer farem una frase de prova. Recorda de repetir tot el que puguis recordar. Estàs a punt?* [First we will do a rehearsal. Remember to repeat everything you hear. Are you ready?]
  The experimenter produces Sentence 1. If the child does not repeat it, the experimenter asks:
  – *Que pots repetir-la?* [Can you repeat it?]
  Otherwise, the experimenter continues:
  – *Molt bé. Ara en farem més de la mateixa manera. Estàs a punt?* [Very good. Now we'll continue the same way. Are you ready?] and sentences up to 60 are repeated.

In the course of the test, the experimenter makes positive, encouraging comments to the child (*Molt bé, continuem!* “Very good! Let's continue!”), independently of how successful his/her repetitions are, at least every 10 items. According to the procedure, the experimenter may give some advice to the child (*Mira de parlar clar, més a poc a poc, para atenció* “Try to speak clearly, a bit more slowly, pay attention”). In principle the child hears each sentence only once, but the sentence may be read a second time if there is a noise or another source of distraction or the child does not repeat the sentence after hearing it the first time. If the child corrects himself/herself, it is the second production that is recorded (whether it is correct or not).

It is relatively standard to ask participants to count up to three in repetition tasks to avoid mere phonological repetition; this request was not made of the children tested, following the procedure of Marinis et al. (2011), who considered that counting up to three would tax the children's memory beyond what is advisable, given the length of the sentences.

### Coding

Two methods can be applied to code the results. The first method consists of coding responses as either correct (1) or incorrect (0), where correct designates a response that is identical to the original (ignoring minor dialectal differences, like for example *meua* “mine” instead of *meva*, since both are well formed in Catalan and one would not expect a speaker of a variety using *meua* to use *meva* when repeating what had been said by a speaker of a variety using *meva*). In this coding method, all responses that are not identical are considered incorrect, even if they are well-formed. This method is widely used for coding SRTs not only because it is easy to apply, but also because it has proven to be very reliable in distinguishing children with SLI from those without (see the discussion in Chiat et al., 2013).

The second method is more sensitive to considerations of grammaticality, as answers are classified into four categories: (i) identical answer; (ii) grammatical but non-identical repetition, exemplified in (5); (iii) ungrammatical answer, exemplified in (6); and (iv) fragment (incomplete, unfinished repetition), exemplified in (7). Under this scoring method, errors are kept separate depending on the structure tested (e.g., errors in wh- interrogatives are scored separately from errors in object clitic production, etc.).

(5) Per què fa el dinar, el pare?
  for what makes the lunch, the father
  “Why is Dad making lunch?”
  (instead of *Per qui fa el dinar, el pare?* “Who is Dad making lunch for?”

(6) No ^*^(l')he vist des de fa deu anys.
  Neg cl3s have-1s seen from 10 years
  “I haven't seen (him) for 10 years.”

(7) Vam decidir anar a la platja.
  PAST1pl decide go to the beach
  (instead of *Vam decidir anar a la platja a nedar* “We decided to go to the beach to swim.”)

## A pilot study with TD children

A pilot study with 30 school-aged children aged 6 and 7 was carried out by Gavarró et al. (2012b). The children were from Sant Cugat and Sabadell, in the metropolitan area of Barcelona, where Central Catalan is spoken. They were recruited in their primary schools, and their parents or tutors gave written consent for testing. All the children were native speakers of Catalan, also speakers of Spanish, since children learn Spanish in the Catalan schooling system (and so bilingual and multilingual children were not excluded), with no hearing or language impairment. They were identified by their teachers as Catalan-dominant. No additional exclusion and inclusion criteria were adopted. Details of the participants appear in Table 2.

**Table 2:** Participants.

	**#**	**Gender**	**Age range**	**Mean age**
6-year-olds	14	10 f, 4 m	5;7,5–6;11,24	6;5,20
7-year-olds	16	10 f, 6 m	7;0,16–8;0,19	7;4,16
Total	30	20 f, 10 m	5;7,5–8;0,19	6;11,14

In what follows I present the results for the whole group and by age. Table 3 summarizes the results under the first scoring method, taking into account whether repetitions were identical or not.

**Table 3:** Raw number and percentage correct repetitions.

	**Identical repetitions**	**Percentage identical(%)**
6-year-olds	748/840	89.05
7-year-olds	855/960	89.06
Total	1603/1800	89.06

Table 4 shows the results obtained when the second scoring method was applied.

**Table 4:** Raw number and percentage of each answer type (identical repetition, grammatical but non-identical repetition, fragment and ungrammatical repetition).

	**Identical repetitions**	**Grammatical repetitions**	**Fragments**	**Ungrammatical repetitions**
6-year-olds	89.05	63/840 (7.5%)	14/840 (1.67%)	15/840 (1.79%)
7-year-olds	89.06	71/960 (7.4%)	4/960 (0.42%)	30/960 (3.13%)
Total	89.06	134/1,800 (7.44%)	18/1,800 (1%)	45/1,800 (2.5%)

The results obtained indicate a high proportion of identical repetitions by the 6- and 7-year-olds for whom the task was designed (0.89 on average, ranging from 0.8 to 0.93 depending on sentence type), and a very low presence of ungrammatical repetitions, as well as a negligible number of fragments.

Let us now examine the results broken down by grammatical structure. Results for 6- and 7-year-olds are given together, given the small difference between the two groups. Table 5 provides the results for level 1 items.

**Table 5:** Answer type, level 1 items.

	**Identical repetition**	**Grammatical repetition**	**Fragments**	**Ungrammatical repetition**
**Finite SVO sentences**				
6- and 7-year-olds	213/240 88.75%	20/240 8.33%	1/240 0.42%	6/240 2.5%
**Third person object clitics**				
6- and 7-year-olds	210/240 87.5%	19/240 7.92%	6/240 2.5%	5/240 2.08%
**Partitive clitics**				
6- and 7-year-olds	110/120 91.67%	1/120 0.83%	0	9/120 7.5%

Table 6 for level 2 items.

**Table 6:** Answer type, level 2 items.

	**Identical repetition**	**Grammatical repetition**	**Fragments**	**Ungrammatical repetition**
**Passives**				
6- and 7-year-olds	223/240 92.92%	9/240 3.75%	3/240 1.25%	5/240 2.08%
**WH- questions**				
6- and 7-year-olds	222/240 92.5%	13/240 5.42%	1/240 0.42%	4/240 1.67%
**Subordinate clauses**				
6- and 7-year-olds	110/120 91.67%	7/120 5.83%	2/120 1.67%	1/120 0.83%

Table 7 provides results for level 3 items.

**Table 7:** Answer type, level 3 items.

	**Identical repetition**	**Grammatical repetition**	**Fragments**	**Ungrammatical repetition**
**Subject relatives**				
6- and 7-year-olds	164/180 91.11%	13/180 7.22%	0/180	3/180 1.67%
**Object relatives**				
6- and 7-year-olds	240/300 80%	47/300 15.67%	5/300 1.67%	8/300 2.67%
**Conditional clauses**				
6- and 7-year-olds	111/120 92.5%	5/120 4.17%	0	4/120 3.33%

The incidence of ungrammatical repetitions was very low, under 3% for all item types except for conditionals (where they amounted to 3.3%) and sentences with partitive clitics. This last case deserves special consideration, as one would expect partitive clitics to be omitted for an extended period in Catalan SLI, as mentioned in section 1. However, there is also some indication in the literature that partitive clitics, under transfer from Spanish in bilingual speakers, may be omitted giving rise to productions that are ungrammatical in monolingual Catalan. Perpiñán (2017) reports that partitive clitic production (8) was judged grammatical by a group of Spanish-dominant bilingual speakers, but partitive omission (9) was also accepted at rates that differed significantly from those of Catalan-dominant bilinguals; likewise, ungrammatical clitic doubling with partitives (10) was accepted more often by Spanish-dominant than Catalan-dominant speakers. The contrast between the two groups was found, and to an even greater extent, in production.

(8) Els bebès sempre tenen gana. El meu sempre *en* té!
  the babies always have hunger the mine always PARTcl has
  “Babies are always hungry. Mine always is!”

(9) (^*^)… El meu sempre té!
  the mine always has

(10) (^*^)… El meu sempre *en* té gana!
  the mine always PARTcl has hunger

Pursuing the same line of research, Gavarró (in press) considered constructions that systematically relate to partitive clitic production in the nominal domain (11) and reached conclusions consistent with those of Perpiñán (2017): depending on the linguistic background of the speaker (Catalan-dominant or Spanish-dominant) partitivity is either overtly marked in the syntax, or it is not, as in contemporary Spanish. Therefore, partitive clitic omission may reflect more the variety of Catalan that the child is exposed to than any risk of language impairment.

(11) La mare porta una maleta gran i una ^*^(de) petita.
  the mother carries a suitcase big and one of small
  “Mother carries a big suitcase and a small one.”

As a consequence, it seemed preferable to suppress partitive clitics from the SRT as a possible source of confound. Likewise, the initial version of the SRT was modified after the pilot study to eliminate some lexical items (*lleter* “milkman,” *abocar* “pour”) that rendered repetition unduly difficult simply because the lexical items were possibly not part of the children's vocabulary. The final version of the SRT involves only 56 items, as the four partitive clitic items were excluded^6^.

Individual results for the initial version of the task can be found in Gavarró et al. (2012b) and are open-access. They show that there is little individual variation; in particular, ungrammatical repetition is low for all the children (with at most one or two errors for the children who produced any error at all). Only one child produced as many as four ungrammatical repetitions out of 60 sentences (a 6.6% error rate).

## Sentence repetition in catalan SLI

The revised version of the SRT for Catalan was administered to five children diagnosed with SLI in Sabadell, Sant Sadurní d'Anoia and Vilanova i la Geltrú, where Central Catalan is spoken. They were all male, their ages ranged from 6;6 to 17;4 (mean age: 10;7), and they were all native speakers of Catalan. Although all of them had knowledge of Spanish and could be considered bilingual, they had been identified as Catalan-dominant by their teachers. They were attending state schools and were undergoing treatment with a speech therapist after having been diagnosed with SLI. They were recruited through CREDA (speech therapy units, initially aimed at children with hearing deficits, run by the Catalan education authority). The intelligence tests administered to them (Wechsler Intelligence Scale for Children-Revised, WISC-R, Wechsler, 1974) indicated scores within the normal range (individual scores n.a.).

The procedure was as described above. The children were tested individually by the author in the schools they were attending, except for one child who was tested at home. The parents or tutors of the participants gave prior written consent to testing, which was conducted following the ethical principles of the Declaration of Helsinki. The session in which the SRT was administered included no further testing, and took between 20 and 30 minutes.

The sample of children tested is small, due to the limited number of children with SLI that could be recruited through CREDA and who also fulfilled the condition of being Catalan-dominant; for this reason, the results for all the participants tested are reported, in spite of age variability. An anonymous reviewer points out that it would have been useful to have fuller information about the participants (both SLI and TD children in the previous study), particularly information on the non-verbal abilities of the participants, type of bilingualism (simultaneous or sequential), length of exposure to Spanish, as well as information on their socio-economic status. However, gaining access to a wider sample and gathering these additional data would have required resources that were not readily available; it remains for future research to avoid both of these shortcomings.

The individual and overall results as obtained under the first scoring method appear in Table 8.

**Table 8:** Identical repetition, raw scores and percentage, SLI children.

	**Identical repetitions**	**Percentage identical(%)**
SLI1	29/56	51.8
SLI2	16/56	28.6
SLI3	15/56	26.8
SLI4	42/56	75
SLI5	32/56	57.1
Total	134/280	47.85

The same data scored according to the second scoring method yielded the results displayed in Table 9.

**Table 9:** Raw number and percentage for each answer type (identical repetition, grammatical but non-identical repetition, fragment and ungrammatical repetition).

	**Identical repetitions**	**Grammatical repetitions**	**Fragments**	**Ungrammatical repetitions**
SLI1	29/56 51.8%	8/56 14.3%	0	19/56 33.9%
SLI2	16/56 28.6%	24/56 42.8%	0	16/56 28.6%
SLI3	15/56 26.8%	28/56 50%	1/56 1.8%	12/56 21.4%
SLI4	42/56 75%	11/56 19.6%	0	3/56 5.4%
SLI5	32/56 57.1%	20/56 35.7%	0	4/56 7.1%
total	134/280 47.85%	91/280 32.5%	1/280 0.35%	54/280 19.28%

Two observations can be made about these results. First, these children with SLI produced a high number of grammatical but non-identical repetitions, and ungrammatical repetitions. Second, there is wide variation within the SLI group: as shown in Table 8, grammatical non-identical repetitions range from 14.3 to 50% and ungrammatical repetitions from 5.4 to 33.9%.

Focusing on the ungrammatical productions, one may wonder in which grammatical constructions children with SLI failed more often. A summary of the errors found appears in Table 10. As can be seen, object relative clauses are the constructions in which more errors are found, and also the single construction in which all the children in our sample fail (object relatives are also the construction in which TD children only succeed in identical repetition in 80% of cases; see Table 7 above). Ungrammatical third person object clitic omission and determiner omission are also found, although in the youngest two children, as well as problems in the repetition of passive sentences. The remaining error types are less common. Determiner agreement errors and determiner omission do not appear in the SRT as separate categories, as determiners are found in all sentence types, but they were relatively common and were therefore tallied separately.

**Table 10:** Error types, children with SLI.

	**SLI1**	**SLI2**	**SLI3**	**SLI4**	**SLI5**	**Total**
Finite verb	2	3				5
Accusative clitic omission	3	3	1			7
Long passive sentences	1	1		1	3	6
Wh- interrogatives			1			1
Complement clause	1		1			2
Subject relative clause		2	2	1		5
Object relative clause	5	4	3	1	1	14
Conditional			3			3
D omission	4	3				7
DP agreement	1		1			2

**Figure 1:**
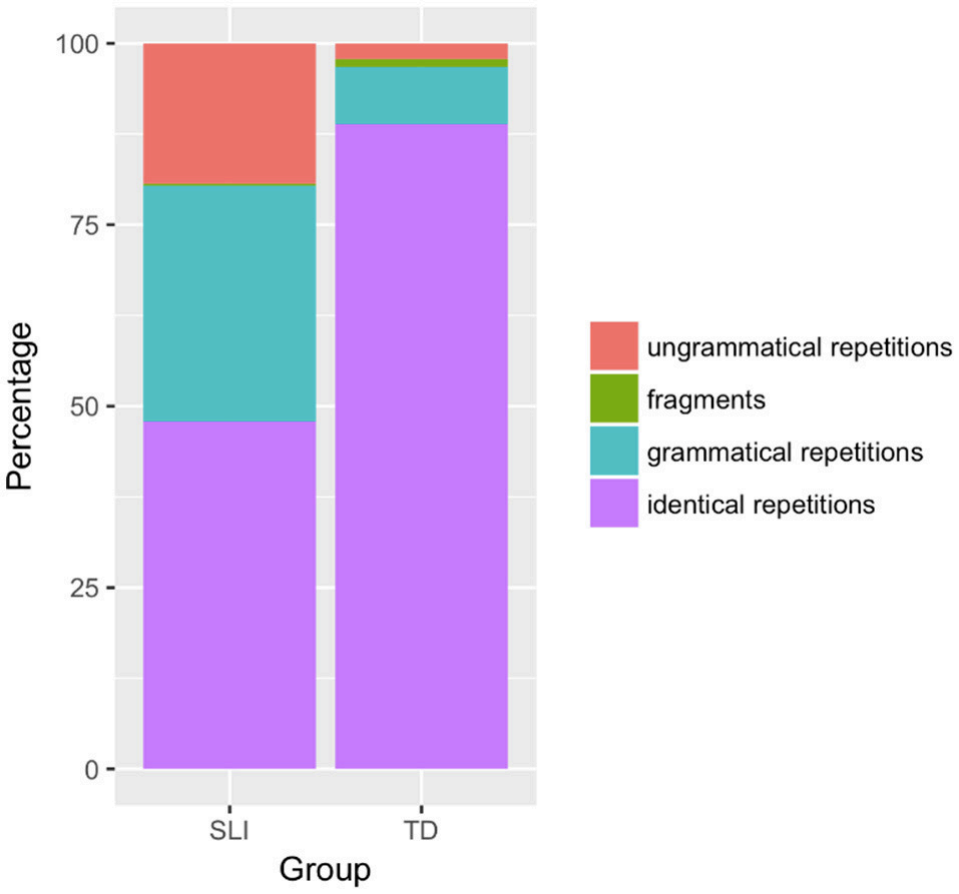
Results, TD and SLI children.

The results for the children with SLI are graphically represented in Figure 1 together with those obtained by the TD group once the partitive clitic items are removed (so that the calculations for both groups are based on 56 rather than 60 items per child).

**Figure 2:**
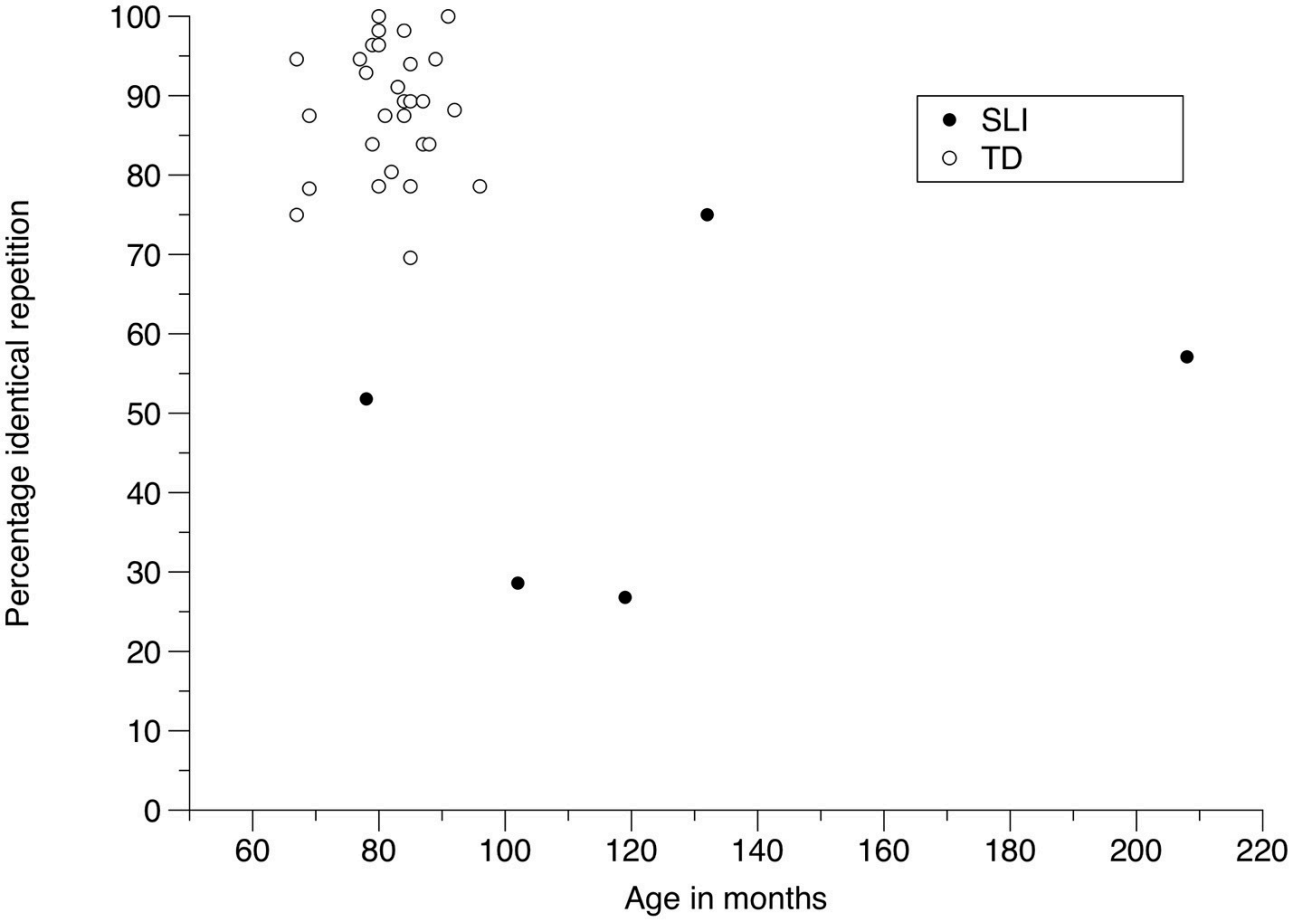
Scatterplot of percentage of identical repetition as a function of age for TD and SLI children.

Because of the small number of subjects in the study, no statistical comparisons were carried out between the results for TD children and children with SLI. The overall scores for the two groups show a contrast: identical repetition occurs in 88.9% of cases for 6- and 7-year-old TD children, but only 47.8% for children with SLI. Under the second scoring method, where error type is taken into account, grammatical but non-identical repetitions amount to 7.9% for TD and 32.5% for SLI children, and ungrammatical repetitions represent 2.1% of answers for TD children but 19.28% for children with SLI (fragments are marginal for both groups: 1.1% for TD children, 0.35% for children with SLI). However, turning to individual performance, one child with SLI, SLI4, produced identical repetitions at a rate of 75%, above the 70% rate of the TD child with the lowest score; another TD child produced a 75% rate of identical repetitions, like SLI4. As a consequence, although at the group level TD and SLI children performed differently, there is overlap in their performance, as shown in Figure 2. The age factor is relevant here: comparing TD and SLI children of the same age, the TD children perform consistently better than the SLI children; the only child with SLI with performance similar to the TD 6- and 7-year-olds is much older. Although some of the 6- and 7-year-old TD children have reached ceiling performance, as a group they have not. Therefore, testing older TD and SLI children would clarify the relation between age and performance on the SRT. With the results available, for clinical purposes, the SRT may be insufficiently accurate in terms of specificity. I consider this further in the discussion.

## Discussion and conclusions

In this section I consider comparable results in the literature on SRTs. Limiting the comparison to the Romance family, SRTs have been designed and administered in several languages closely related to Catalan. In Italian, Devescovi and Caselli (2007) designed a task aimed at children as young as 2; their goal was not to discriminate between TD children and children at risk or with SLI, but to observe language development by means of repetition rather than production. Given the different nature of their goals and participants with respect to those of the present study, I do not pursue a comparison of the results. A more accurate comparison is possible with the LITMUS SRT designed for French, also under the auspices of COST IS0804, as described in Fleckstein et al. (2016). The sentence types tested in French were (i) finite clauses in the present tense, (ii) finite clauses in the past tense, (iii) object wh- questions, (iv) finite and non-finite complement clauses, and (v) subject and object relative clauses. There is therefore considerable overlap between structures covered by their SRT and those put forward here, although in the French SRT more emphasis is placed on verbal finiteness (French being a non-null subject language). Fleckstein et al. (2016) tested 37 monolingual TD children (aged 5;7 to 6;5) and 13 monolingual children with SLI (aged 6;11 to 8;4), as well as bilingual children. Their results appear in Table 11. I include the results for monolingual and bilingual children, although I would argue that the Catalan-speaking children in the sample here are closer in profile to the monolinguals than the bilinguals, given that they are Catalan-dominant.

**Table 11:** Percentage of identical repetition by monolingual (Mo) and bilingual (Bi) TD and SLI children, French (Fleckstein et al., 2016).

**Structure subtype**	**Mo-TD (%)**	**Bi-TD (%)**	**Mo-SLI (%)**	**Bi-SLI (%)**
Present 3sg	98.2	96.2	76.9	75
Present 3pl	95.5	82.9	48.7	38.9
Past 3sg	98.2	90.5	64.1	63.9
Past 3pl	95.5	76.2	43.6	16.7
Who question	100	100	76.9	80.6
Which question	96.4	94.3	56.4	58.3
Non-finite complement	84.7	70.5	30.8	27.8
Finite complement	75.7	53.3	10.3	5.6
Subject relative	96.4	80.9	51.3	33.3
Object relative	89.2	65.7	25.6	19.4

Monolingual TD children scored very high, generally higher than the Catalan TD children, even though their ages are similar. In any event, TD children in both language groups achieved over 90% identical repetition for all the sentence types tested except, in French, non-finite and finite complement clauses and object relatives and, in Catalan, finite clauses, third person object clitics, and object relative clauses. The contrast in performance with the children with SLI is evident for all sentence types. Comparing the results in Table 11 with those for Catalan, object relatives stand out as the one construction which cross-linguistically shows the effects of SLI (object clitic production was not included in the SRT in French in spite of it being delayed in SLI).

Fleckstein et al. (2016) tested 47 bilingual children (French-Arabic, French-English): 35 with typical development, 12 with SLI. Overall, TD bilinguals and monolinguals showed identical repetition rates of 81 and 93%, respectively, and SLI bilinguals and monolinguals showed rates of 41.9 and 48.5%, respectively (compared to results on the Catalan SRT of 88.9% for TD and 47.85% for SLI children). The difference in performance between the four groups was statistically significant. Regarding individual performance, the LITMUS-SRT developed for French had, for monolinguals, a specificity of 91.9% and a sensitivity of 92.3%. For bilinguals, measures for both specificity and sensitivity were lower, but still above 80%, a rate which is considered acceptable by Plante and Vance (1994).

The results for Catalan are much more limited than those for French, but, together with the quantitative resemblance, they bear a promising similarity to those of French in two respects: the ability to distinguish TD from SLI children, and the ease with which they can be obtained: an SRT for which identical vs. non-identical repetition is computed. The facility in performing and scoring the task is an advantage for participants and for the professionals involved, and a simple scoring method also makes results more reliable (compare this method to that required to score different error types, which necessitates highly trained clinicians and is likely to give rise to many more dubious cases).

With the partial results available at present one can observe overlap in the performance of the Catalan-speaking children with TD and SLI, even if, as pointed out, the child with SLI with the highest score (75% identical repetition) was older by more than 3 years than the oldest TD child in the study. The children with SLI matched in age with the 6- and 7-year-old groups performed worse and there was no overlap in performance between TD and SLI participants. Despite the scarcity of studies of SLI in adulthood (Stothard et al., 1998; Clegg et al., 2005), there is evidence that the linguistic behavior of individuals with SLI varies with age, quantitatively and qualitatively (see above, for the case of third person object clitics through childhood, Gavarró, 2012; Arosio et al., 2014). As already indicated, testing a broader age range and conducting a proper comparison of age-matched groups of TD and SLI children remains for future research^7^.

The next step is for the SRT for Catalan proposed here to be normalized and run with a large number of children with SLI. Only then will it be possible to take measures of sensitivity and specificity. At a later stage, testing with late bilingual children should be undertaken. Caution is necessary since no experimental work on Catalan L2 tells us if the constructions in the SRTs are vulnerable in L2 (or in the L2 of a subset of children, depending on their L1). For example, in the case of third person object clitics, there is evidence from other, related languages that omission may also be found in L2 (see Grüter, 2005 for French), although the source of this omission would be different in nature from that seen in TD young children and SLI (transfer from a null object language, etc.).

The two groups for whom results have been reported here were bilingual (or multilingual) to varying extents. Our inclusion criterion was that they should be raised natively in Catalan, but there is the possibility that one of their parents raised them as native speakers of another language. The Catalan schooling system implements immersion in Catalan, but Spanish is also taught as part of the curriculum and children have plenty of opportunity to be exposed to Spanish through the media, acquaintances, friends and relatives. In this kind of context it is difficult to control for the kind of linguistic exposure that children get in terms of AoA, LoE, etc., although it should still be possible by means of a parental questionnaire.

One of the issues addressed in this topic is whether bilingual advantage (Bialystok et al., 2012) is attested in children like those in our sample, and whether it is detectable in both TD and SLI children alike. Bilingual advantage is argued to play a role in receptive and expressive vocabulary, verbal working memory and executive function in general. These areas, therefore, are the ones in which bilingual advantage would be predicted for TD and SLI bilinguals. The grammatical domain on which this paper focuses, on the other hand, appears to be orthogonal to bilingual advantage. When we set out to characterize how SLI manifests itself in Catalan speakers, we aim at core syntactic features (or phonological features, for phonological SLI) that are affected in SLI and remain unaltered due to an underlying disorder. To my knowledge no study so far has claimed that syntactic features or operations are subject to bilingual advantage. While bilingualism affects cognition and has a neurological impact, the core syntactic features of SLI seem to remain constant and depend mainly on the grammatical features of the language acquired, as we have shown.

To summarize, I have provided an SRT strongly motivated in our current knowledge of SLI in Catalan and closely related languages, mostly French and Italian for third person object clitic constructions, Italian for finiteness, languages with post-nominal relatives for relatives, and languages with verbal passives for passives. The results so far are just a first step, but indicate that the task has the potential to serve as a reliable and efficient tool to discriminate between TD children and children with SLI.

## Author contributions

I am the sole author of the paper. Former work in collaboration with other people is duly referenced.

### Conflict of interest statement

The author declares that the research was conducted in the absence of any commercial or financial relationships that could be construed as a potential conflict of interest.

## Footnotes

^1^The problem of misdiagnosis is not new: it is extensively documented for the immigrant population reaching the US in the first half of the Twentieth century—and on that occasion children were often misdiagnosed as mentally retarded (see Romaine, 1989).

^2^In more contemporary terms, instances of uninterpretable feature elimination.

^3^For Spanish there exists a non-word repetition task consisting of twenty pseudowords, four of each syllable length, one to five (Girbau and Schwarz, 2008; Girbau, 2016). (See also Aguado et al., 2006). To my knowledge, no such tool exists for Catalan, although a screening test which was being developed for Catalan, GAPS (van der Lely et al., 2010) included a short non-word repetition task with two monosyllabic control items and eight test items of increasing difficulty, from one to four syllables.

^4^Vinther (2002) argues that for grammatical analysis to be necessarily involved in sentence repetition, sentences should be longer than 6–8 syllables.

^5^In addition to the contrast between subject relatives and object relatives, there is a contrast between subject-modifying and object-modifying relative clauses, where the subject-modifying clauses are easier to process than the object-modifying ones (see Stavrakaki et al., 2015 for a summary of the findings). Subject-modifying and object-modifying relatives were equally represented in the subject relative and object relative items.

^6^The complete revised version of the SRT can be found at: http://filcat.uab.cat/clt/publicacions/reports/pdf/GGT-12-02.pdf.

^7^Girbau (2016), in her non-word repetitition task, tested a group of children of ages 8;0 to 9;11, and to my knowledge no study on Catalan SLI goes beyond that age.
